# Vestibular dose correlates with dizziness after radiosurgery for the treatment of vestibular schwannoma

**DOI:** 10.1186/s13014-021-01793-7

**Published:** 2021-03-26

**Authors:** Ekin Ermiş, Lukas Anschuetz, Dominic Leiser, Robert Poel, Andreas Raabe, Peter Manser, Daniel M. Aebersold, Marco Caversaccio, Georgios Mantokoudis, Janine Abu-Isa, Franca Wagner, Evelyn Herrmann

**Affiliations:** 1 Department of Radiation Oncology, Inselspital, Bern University Hospital, University of Bern, Freiburgstrasse 18, 3010 Bern, Switzerland; 2 Department of Otorhinolaryngology, Head and Neck Surgery, Inselspital, Bern University Hospital, University of Bern, Bern, Switzerland; 3 Center for Proton Therapy, Paul Scherrer Institute, Villigen, Switzerland; 4 Department of Neurosurgery, Inselspital, Bern University Hospital, University of Bern, Bern, Switzerland; 5 Division of Medical Radiation Physics, Department of Radiation Oncology, Inselspital, Bern University Hospital, University of Bern, Bern, Switzerland; 6 Department of Diagnostic and Interventional Neuroradiology, Inselspital, Bern University Hospital, University of Bern, Bern, Switzerland; 7 Department of Radiation Oncology, Hôpital Riviera-Chablais, Rennaz, Switzerland

**Keywords:** Vestibular schwannoma, Vestibule, Radiosurgery, Dizziness

## Abstract

**Background:**

Stereotactic radiosurgery (SRS) has been recognized as a first-line treatment option for small to moderate sized vestibular schwannoma (VS). Our aim is to evaluate the impact of SRS doses and other patient and disease characteristics on vestibular function in patients with VS.

**Methods:**

Data on VS patients treated with single-fraction SRS to 12 Gy were retrospectively reviewed. No dose constraints were given to the vestibule during optimization in treatment planning. Patient and tumor characteristics, pre- and post-SRS vestibular examination results and patient-reported dizziness were assessed from patient records.

**Results:**

Fifty-three patients were analyzed. Median follow-up was 32 months (range, 6–79). The median minimum, mean and maximum vestibular doses were 2.6 ± 1.6 Gy, 6.7 ± 2.8 Gy, and 11 ± 3.6 Gy, respectively. On univariate analysis, Koos grade (*p* = 0.04; OR: 3.45; 95% CI 1.01–11.81), tumor volume (median 6.1 cm^3^; range, 0.8–38; *p* = 0.01; OR: 4.85; 95% CI 1.43–16.49), presence of pre-SRS dizziness (*p* = 0.02; OR: 3.98; 95% CI 1.19–13.24) and minimum vestibular dose (*p* = 0.033; OR: 1.55; 95% CI 1.03–2.32) showed a significant association with patient-reported dizziness. On multivariate analysis, minimum vestibular dose remained significant (*p* = 0.02; OR: 1.75; 95% CI 1.05–2.89). Patients with improved caloric function had received significantly lower mean (1.5 ± 0.7 Gy, *p* = 0.01) and maximum doses (4 ± 1.5 Gy, *p* = 0.01) to the vestibule.

**Conclusions:**

Our results reveal that 5 Gy and above minimum vestibular doses significantly worsened dizziness. Additionally, mean and maximum doses received by the vestibule were significantly lower in patients who had improved caloric function. Further investigations are needed to determine dose-volume parameters and their effects on vestibular toxicity.

## Background

Sterotactic radiosurgery (SRS) is considered as a first-line treatment for small or moderate sized vestibular schwannomas (VS) [1–4]. However, its impact on vestibular function and perception of dizziness depending on the applied dose remains uninvestigated. Despite their relatively slow growth, some patients with VS present with symptoms including hearing loss (about 90%), tinnitus (65–75%), impaired balance (about 60%) and neuropathies of the cranial nerves (4–8%) [5]. These presenting symptoms and/or continued expansion of the tumor may lead clinicians to seek for the optimal management option. The toxicity profile of each intervention, along with the patient and tumor characteristics, should be considered in decision-making on management [6, 7].

Much research has been published on post-treatment morbidity related to radiotherapy [8–10]. So far, most of the studies analyzing treatment toxicity have focused primarily on hearing impairment and cranial nerve damage. The mechanism of immediate and delayed hearing deterioration has been scrutinized [11, 12]. In relation to hearing loss, the radiation dose to the cochlea and its components (e.g. modiolus), to the vestibulocochlear nerve, and the cochlear nucleus in the brain has been measured leading to dose-volume recommendations [13–15]. Cranial nerve outcomes have been reported and optimal marginal doses for better nerve preservation have been proposed [9, 16]. However, data on the effects of radiotherapy, especially SRS, on dizziness are scarce. Vestibular toxicity can be defined according to the common terminology criteria for adverse events version 5.0 as a disorder characterized by dizziness, imbalance, nausea, and vision problems [17]. Dizziness is the sensation of disturbed or impaired spatial orientation without a false or distorted sense of motion [18]. It could be the main presenting symptom and can be extremely disturbing with deterioration on the patient’s quality of life [19, 20]. Previous studies showed that after SRS, about 17% of patients developed transient dizziness and up to 3% had continuous (chronic) dizziness [21, 22]. Given that vestibular toxicity is a strong predictor of the quality of life of VS patients, research evaluating this parameter would contribute to preserving health-related well-being [23, 24].

To the best of our knowledge, none of the published studies has shown statistically relevant results relating the radiotherapy dose to the vestibule to changes in dizziness after SRS. The primary objective of the current study was to evaluate the impact of SRS doses and other patient and disease characteristics on dizziness in patients with VS. Such information should help to define vestibular dosimetric parameters in patients treated with SRS and may assist in determining the vestibular dosimetry and its relationship to dizziness.

## Methods

### Patient selection

With the approval of the regional ethics committee, patients who received SRS for VS between 2010 and 2016 were retrospectively evaluated.

Fifty-three patients met the inclusion criteria of receiving single-fraction SRS for unilateral, sporadic VS. Patients < 18 years old, with neurofibromatosis type 2, those who had undergone prior radiotherapy or microsurgery, and patients who were treated with multi-fractionation schemes were excluded from the study. Clinical and radiosurgical data were collected from patient charts and medical follow-up notes, including: age, sex, tumor location and morphology, pre-SRS growth, tumor volume, Koos grade, pre-SRS dizziness/tinnitus, target and vestibule doses (minimum, mean and maximum), post-SRS transient volume expansion (defined as volume growth followed by shrinkage to the pre-SRS volume or less), post-SRS tumor necrosis (detected by sequential magnetic resonance (MR) images) and objective neurootological examination.

### Radiosurgical procedure

All patients received treatment either with a Linac (Novalis TX, BrainLAB AG, Feldkirchen, Germany; Varian Medical Systems, Palo Alto, CA, USA) or robotic-based (Cyberknife, Accuray, Sunnyvale, California) radiosurgical system. Patients were immobilized in the supine position using a commercial stereotactic mask fixation system. Thin-sliced (0.75 mm) computed tomography (CT) images without contrast were obtained. Contrast-enhanced T1- and T2-weighted high-resolution MR images (1 mm) and, when available, three-dimensional constructive interference in steady-state (3D-CISS) sequence images were registered with CT images for target delineation. After tumor delineation by a radiation oncologist, a medical physicist planned the treatment. No additional margin to the target volume was included for the planning target volume. A single fraction of 12 Gy to the mean 78% (range, 52–90%) isodose line for Cyberknife treatment plans and to the mean 94% (range, 85–98%) isodose line for Novalis treatment plans was prescribed. SRS plans were generated and delivered using the treatment planning systems of iPlan (Novalis) and Multiplan (Cyberknife). The quality of the treatment plans was assessed by evaluating target coverage, dose heterogeneity/conformity, and normal tissue dose tolerance, particularly cochlear dosimetry. KV imaging (ExacTrac, Brainlab) for Linac-based and real-time X-ray patient tracking (6D-Skull) for robotic-based systems were used for set-up verification and repositioning.

### Patient follow-up

All patients were followed up at 6 months using MR imaging and clinical examination and again at 1 year post-SRS with additional objective neurootological tests. Long-term assessments were performed annually. Vestibular testing comprised a patient history, clinical neurotological and focused neurological examination including cranial nerves III-XII, cerebellar examination, stance tests (Romberg [standing > 20 s] and Unterberger [walking 50 steps in place without deviation > 45°]). The vestibular tests were consist of video-oculography including head hanging test (Rose manoeuvre), test for spontaneous nystagmus at all gaze directions, smooth pursuit, rotatory chair examination (sinusoidal harmonic acceleration [SHA] test at 60 °/s chair velocity and 0.02 Hz), cervical vestibular evoked myogenic potentials (cVEMP, 500-Hz tone-burst stimuli) and bithermal caloric testing. The degree of asymmetry in caloric testing was categorized in; normal (0–25%), mild hypofunction (26–50%), moderate hypofunction (51–75%) and severe hypofunction (76–100%). Clinical examination and vestibular test results were reported pre-SRS and 1-year post-SRS. The results were classified as stable/improved if the patient maintained the same function or showed better function, and as worse if the function deteriorated. Patient-reported dizziness was recorded pre-SRS and at 6 months post-SRS. At each time point, patients were asked whether they experienced any form of dizziness (at the time of the examination, or any episode) and the responses were coded as a binary outcome, yes or no.

### Dosimetry of the vestibule

**Figure1:**
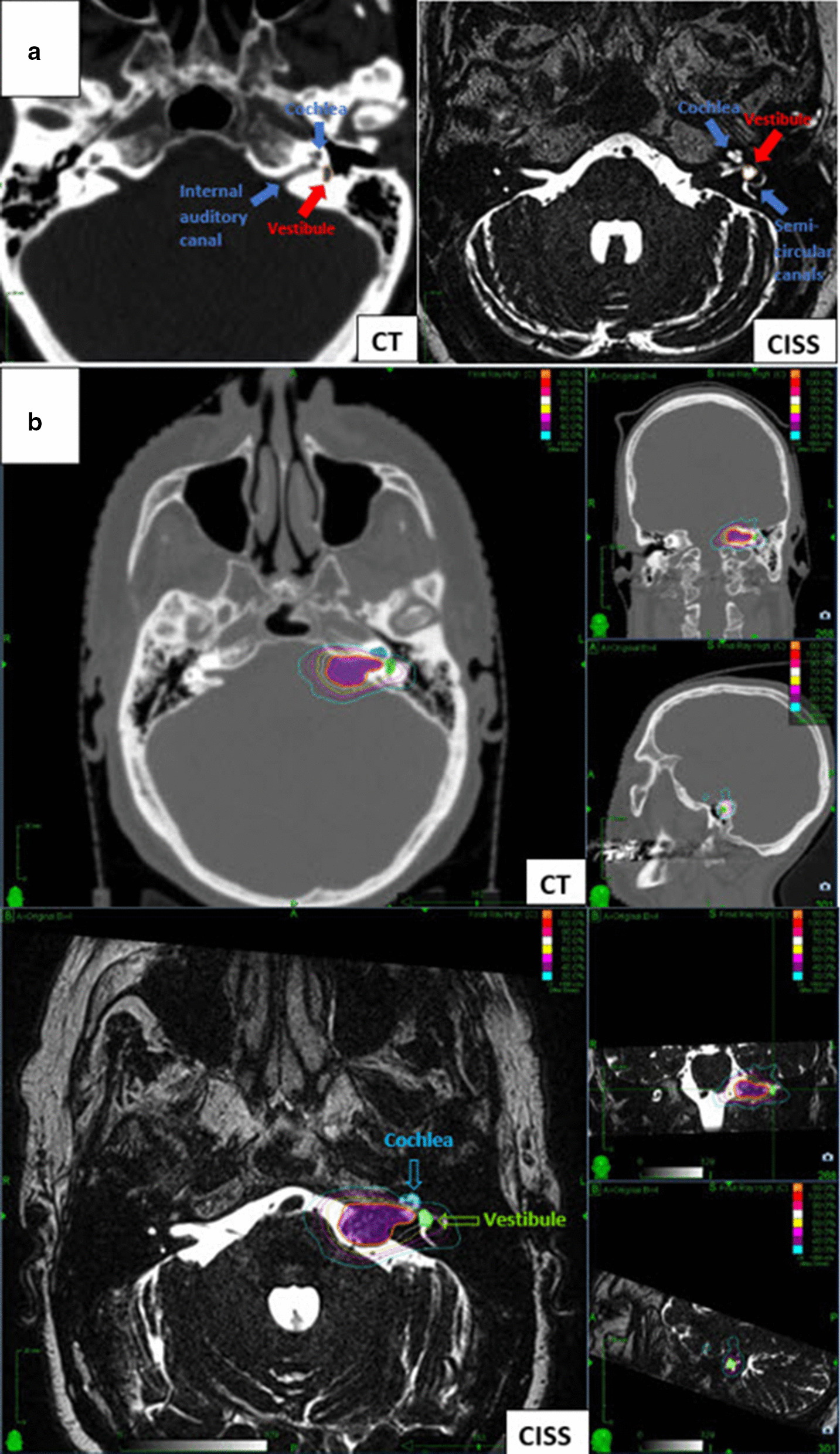
**a** Axial CT (left) and CISS images showing the volume of vestibule (red arrow, orange line). The blue arrows indicate cochlea, semi-circular canals and internal auditory canal. *CT* computed tomography, *CISS* constructive interference in steady-state. **b** Axial, sagittal and coronal CISS MR and CT images showing the volume of the cochlea (blue arrow) and vestibule (green arrow) with the different isodose lines from a treatment plan for Cyberknife. A dose of 12 Gy in a single fraction was prescribed to 80% isodose line and no dose constraints were given to the vestibule during treatment plan optimization. It is evident that cochlea is very well spared by giving it priority during plan optimization, while 70% (10.5 Gy) to 30% (4.5 Gy) isodose lines cover the vestibular volume

Based on the CT and MR images for each SRS plan, the volume of the vestibule was defined with the assistance of a senior board-certified neuroradiologist specialized in head and neck imaging (FW). Six to ten successive axial slices were used for precise volume definition. Location of the vestibule was defined as the common junction of the cochlea and semi-circular canals, where internal auditory canal separates cochlea from the vestibule (Fig. 1a). The minimum, mean, and maximum radiation doses received by the vestibule volume were obtained from the treatment planning software. Vestibule delineation was performed retrospectively and no dose constraints were given to the vestibule during plan optimization.

### Statistics

A certified statistician (DL) performed the statistical analysis. Duration of follow-up was defined as starting from the day of SRS. Descriptive analyses were reported as mean (standard deviation), median (range) or number (percentage). Logistic regression was used to assess variables that might influence dizziness at the specified follow-up times. Variables with a *p *value < 0.1 in the univariate analyses were included in the multivariate analysis. The t-test was used to test the association between continuous variables.

A *p* value of < 0.05 was set as statistically significant. Statistical analyses were performed with IBM SPSS Statistics software (version 25; IBM Corp., Armonk, NY, USA).

## Results

### Demographics

The study cohort consisted of 27 male (51%) and 26 female (49%) patients. The median follow-up duration was 32 months (range, 6–79 months). Table 1 summarizes baseline patient and VS characteristics. Thirty-three patients (62%) were treated with the Novalis TX system. The remaining twenty patients received SRS with the Cyberknife system, after it was introduced in the facility in 2014. The mean age at the time of the SRS was 60 years (range, 23–80 years). The median target volume was 6.18 cm^3^ (range, 0.8–38 cm^3^). Thirty-two patients (60%) had lower Koos grade (I–II) and 21 patients (40%) had higher Koos grade (III–IV). Initially, 37 patients (70%) had tinnitus and the same number reported dizziness. Clinical examination showed that six patients (11%) had spontaneous nystagmus, seven (13%) and 14 (26%) abnormal Romberg and Unterberger tests, respectively. Among vestibular tests, in 16 patients (30%) cVEMP responses were absent before treatment and the median percentage caloric weakness was 33.5% (range, 3–100%).

**Table 1:** Baseline patient and tumor characteristics, n = 53

Characteristic	Value^a^
Age (years)	60 (23–80)
Sex	
Male	27 (51)
Female	26 (49)
Location	
Intra-canalicular	10 (19)
Extra-canalicular	43 (81)
Cystic component	8 (15)
Target volume (cm^3^)	6.18 (0.8–38)
Pre-SRS tumor growth	36 (68)
Koos grade	
I	7 (13)
II	26 (49)
III	11 (21)
IV	10 (17)
Pre-SRS tinnitus	37 (70)
Patient-reported dizziness	37 (70)
Clinical examination	
Spontaneous nystagmus	6 (11)
Romberg test	
Normal	31 (58)
Abnormal (positive)	7 (13.5)
Unterberger test	
Normal	24 (45)
Abnormal (positive)	14 (26.5)
No data available	15 (28.5)
Vestibular tests	
Video-oculography	
No spontaneous nystagmus	27 (51)
Spontaneous nystagmus	4 (7.5)
Pathologic smooth pursuit	7 (13)
No data available	15 (28.5)
Caloric ips. testing (degree of asymmetry)	
Normal (0–25%)	3 (5)
Mild hypofunction (26–50%)	29 (55)
Moderate hypofunction (51–75%)	2 (4)
Severe hypfunction (76–100%)	4 (7.5)
Percentage caloric weakness (%)	33.5 (3–100)
No data available	15 (28.5)
cVEMP	
Normal	8 (15)
Absent reflex	16 (30)
No data available	29 (55)

*cVEMP* cervical vestibular evoked myogenic potentials, *SRS* stereotactic radiosurgery, *ips.* ipsilateral

^a^Values are median (range) or number (percentage)

### Dose parameters, dizziness and vestibular outcome

The mean target volume dose for the entire cohort, Cyberknife and Novalis delivery systems were 12.7 Gy (range, 11.8–17.7 Gy), 13.8 (range, 12.6–17.7 Gy) and 12.4 Gy (range, 11.8–13.4 Gy), respectively. The median, minimum, mean, and maximum radiation doses delivered to the vestibule for the entire cohort were 2.6 Gy (range, 0.05–6.2 Gy), 6.7 Gy (range, 0.3–10.8 Gy) and 11 Gy (range, 2–17 Gy), respectively. For the Cyberknife delivery system, detected vestibular doses were minimum 2.9 Gy (range, 0.05–4.7 Gy), mean 5.9 Gy (range, 0.3–9.1 Gy) and maximum 9.3 Gy (range, 2.1–17.1 Gy). For the Novalis delivery system, dose calculations on the vestibule were minimum 2.8 Gy (range, 0.2–6 Gy), mean 7.2 Gy (range, 0.5–10.8 Gy) and maximum 11.8 Gy (range, 2–13.7 Gy). Post-SRS, eighteen patients (34%) experienced transient volume expansion. In 31 patients (58%), radiological tumor necrosis was detected. At the 6-month follow-up visit, 40 patients (75.5%) reported stable or improved dizziness, while in eight patients (15%), dizziness had worsened. There was a correlation between caloric testing worsening and patient reported dizziness worsening in three out of eight patients. No data were available for five patients (9.5%).

Vestibular testing at 1 year showed that results of video-oculography were stable/improved in 24 patients (45%). The median percentage caloric weakness increased to 76% (range, 2–100%). Twelve patients showed a stable/improved caloric test result in relation to the categories regarding the degree of asymmetry, while 13 patients had a worsened low frequency function. Following treatment, cVEMP responses were present in seven patients, with a stable/improved response rate of 24.5%. Post-SRS tumor characteristics, outcome of dizziness, results of the clinical examination and vestibular tests, and the dose parameters of SRS are summarized in Table 2. Figure 1b represents an example case with the dose distributions in a Cyberknife treatment plan.

**Table 2:** Dose parameters and vestibular outcome after SRS, n = 53

Variable	Value^a^
Vestibule doses (Gy)	
Minimum	2.6 (0.05–6.2)
Mean	6.7 (0.3–10.8)
Maximum	11 (2–17)
Target volume doses (Gy)	
Minimum	11.3 (6.2–12)
Mean	12.7 (11.8–17.7)
Maximum	13 (12.1–23)
Transient volume expansion	18 (34)
Post-SRS tumor necrosis	31 (58)
Patient-reported dizziness	
Stable/improved	40 (75.5)
Worsened	8 (15)
No data available	5 (9.5)
Clinical examination	
Spontaneous nystagmus	
Stable/improved	29 (55)
Worsened	9 (17.5)
No data available	15 (28.5)
Romberg test	
Stable/improved	27 (51)
Worsened	2 (4)
No data available	24 (45)
Unterberger test	
Stable/improved	19 (36)
Worsened	10 (19)
No data available	24 (45)
Vestibular tests	
Video-oculography	
No spontaneous nystagmus	22 (42)
Spontaneous nystagmus	3 (5)
Pathologic smooth pursuit	3 (5)
No data available	25 (48)
Stable/improved	24 (45)
Worsened	2 (4)
Caloric ips. testing	
Normal (0–25%)	2 (4)
Mild hypofunction (26–50%)	9 (17.5)
Moderate hypofunction (51–75%)	3 (5)
Severe hypofunction (76–100%)	11 (21)
Percentage caloric weakness (%)	76 (2–100)
No data available	28 (52.5)
Stable/improved	12 (23)
Worsened	13 (24.5)
cVEMP	
Normal	7 (13)
Absent reflex	14 (26)
No data available	32 (60.5)
Stable/improved	13 (24.5)
Worsened	2 (4)

*cVEMP* cervical vestibular evoked myogenic potentials, *SRS* stereotactic radiosurgery, *ips.* ipsilateral

^a^Values are median (range) or number (percentage)

### Risk factors for dizziness

**Fig. 2:**
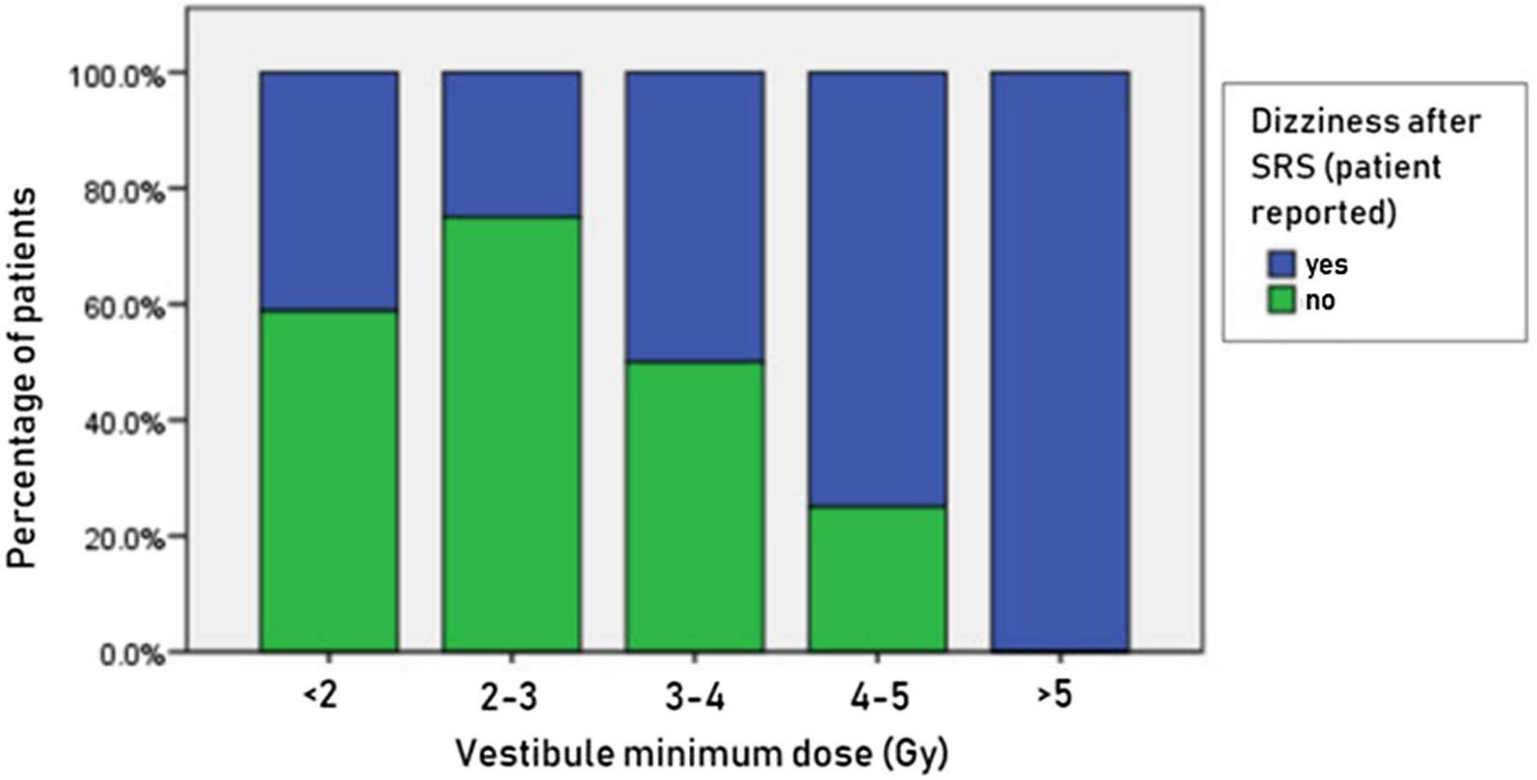
Graph showing the correlation between minimum radiation dose levels to the vestibule and percentage of patients reporting dizziness

Univariate analyses showed that larger target volume (> 6.1 cm^3^; *p* = 0.01; OR: 4.85; 95% CI 1.43–16.49), higher Koos grade (III–IV vs I–II; *p* = 0.04; OR: 3.45; 95% CI 1.01–11.81), presence of pre-SRS dizziness (*p* = 0.02; OR: 3.98; 95% CI 1.19–13.24) and minimum dose to the vestibule (*p* = 0.03; OR: 1.55; 95% CI 1.03–2.32) were significantly associated with patient-reported dizziness after SRS (Table 3). In the subsequent multivariate analyses, minimum dose to the vestibule (*p* = 0.02; OR: 1.75; 95% CI 1.05–2.89) remained significantly associated with dizziness. Figure 2 relates various minimum dose levels received by vestibule to dizziness after SRS. For the patients treated with a minimum dose of < 2 Gy, 2–3 Gy, 3–4 Gy, 4–5 Gy, and > 5 Gy, dizziness rates of 40%, 25%, 50%, 75% and 100% respectively, were found. Notably, all patients who received a minimum vestibular dose of more than 5 Gy reported dizziness after treatment.

**Table 3:** Association between patient, disease, and dose characteristics, and patient-reported dizziness

Parameter	Univariate variables		Multivariate model	
	OR (95% CI)	*p* value	OR (95% CI)	*p *value
Age in years (> 65 vs ≤ 65)	1.12 (0.32–3.90)	0.85		
Location (intra- vs extra-canalicular)	2.10 (0.46–9.64)	0.33		
Cystic component (yes vs no)	1.10 (0.24–5.04)	0.89		
Target volume (> vs ≤ 6.1 cm^3^)	4.85 (1.43–16.49)	**0.01**	2.84 (0.53–15.04)	0.21
Target minimum dose	0.85 (0.27–2.67)	0.80		
Target mean dose	0.72 (0.23–2.24)	0.57		
Target maximum dose	0.51 (0.16–1.61)	0.25		
Pre-SRS tumor growth (yes vs no)	2.02 (0.56–7.31)	0.28		
Koos grade (III–IV vs I–II)	3.45 (1.01–11.81)	**0.04**	2.41 (0.41–14.24)	0.33
Pre-SRS tinnitus (yes vs no)	1.33 (0.38–4.67)	0.65		
Pre-SRS dizziness (yes vs no)	3.98 (1.19–13.24)	**0.02**	4.15 (1.00–17.20)	0.05
Vestibule minimum dose	1.55 (1.03–2.32)	**0.03**	1.75 (1.05–2.89)	**0.02**
Vestibule mean dose	1.10 (0.89–1.35)	0.35		
Vestibule maximum dose	1.01 (0.86–1.19)	0.82		
Transient volume expansion (yes vs no)	1.96 (0.58–6.61)	0.27		
Novalis vs Cyberknife	0.47 (0.14–1.55)	0.21		

Values in bold statistically significant

*CI* confidence interval, *OR* odds ratio, *SRS* stereotactic radiosurgery

The *t* test showed that patients with improved caloric function after SRS had received significantly lower mean (1.5 ± 0.7 Gy, *p* = 0.01) and maximum doses (4 ± 1.5 Gy, *p* = 0.01) to the vestibule than the patients who had a stable or worsened caloric function. Table 4 summarizes dosimetric parameters of the vestibule related to caloric function outcome. No other significant correlation could be found between vestibular test results and patient, disease, or dose characteristics.

**Table 4:** Summary of dosimetric parameters of the vestibule related to caloric function outcome

Parameter	Caloric function outcome after SRS					
	Stable	*p *value^a^	Improved	*p* value^a^	Worsened	*p* value^a^
No of patients	9	–	3	–	13	–
Min. dose to vestibule (Gy)^b^	2.6 ± 1.5	–	0.46 ± 0.2	0.06	3 ± 1.2	0.52
Mean dose to vestibule (Gy)^b^	6.9 ± 2.8	–	1.5 ± 0.7	**0.01**	6.6 ± 1.7	0.79
Max dose to vestibule (Gy)^b^	11.3 ± 3.7	–	4 ± 1.5	**0.01**	10.2 ± 2.1	0.41

Values in bold statistically significant

^a^T-test

^b^Values are mean ± standard deviation

## Discussion

The effectiveness of an intervention in treating a benign disease can be assessed, in addition to the control of the disease, by its impact on the patient’s daily life. Dizziness affects 30–65% of patients diagnosed with VS, regardless of the treatment modality. Patients would like to know whether the proposed intervention is expected to relieve their most significant symptoms [25]. Several studies have demonstrated that dizziness is a more effective predictor of patient-reported quality of life than other parameters such as hearing loss or facial neuropathy [23–25]. Certainly, more research is warranted to better understand the mechanism and management of the dizziness. On the other hand, there is no direct correlation between vestibular function and dizziness perception reported in the literature. Dizziness perception is rather dependent on central vestibular compensation mechanisms. Vestibular compensation and adaptation starts immediately after a lesion on the vestibular nerve [26]. However, preserving or even improving vestibular function after SRS in combination with vestibular physiotherapy has the potential to improve the quality of life of these patients [27]. Our study focused mainly on whether radiation doses to the vestibule have an impact on patient-reported dizziness and vestibular test results.

The European Particle Therapy Network consensus, published in 2018, includes recommendations on delineation of radiation sensitive organs at risk and their dose constraints for an unbiased comparison of different radiation modalities and techniques [28]. In the section on vestibulum and semi-circular canals, the authors noted that few data were available on vestibular toxicity and radiotherapy and emphasized the importance of prospective collection of dose-volume data and accurate toxicity scoring. Stavas et al. conducted one of the few studies on this subject. The authors studied how the vestibule dose might predict change in balance function and patient-reported dizziness after SRS in a prospective observational pilot study [29]. Of the ten patients included, nine were treated with fractionated stereotactic radiotherapy (20–22 Gy in five fractions) and one with SRS (12 Gy). Dose volume data were obtained including mean and maximum dose to the vestibule and the volume of vestibule receiving 5 Gy, 10 Gy and 15 Gy (V5, V10, and V15). Self-reported measures were evaluated with Dizziness Handicap Inventory (DHI). No significant associations or identifiable trends were found between the radiation dose and the change in objective or subjective vestibular function. A related study was presented as a poster at the 37th European Society of Radiotherapy and Oncology congress by Bambery and Cameron. They investigated the relationship between labyrinth dose and dizziness in VS patients [30]. Data on 114 patients treated with SRS with a mean dose of 12.3 Gy were retrospectively reviewed. Labyrinth and vestibule were retrospectively contoured on all treatment plans and length of vestibular nerve treated with treatment dose measured. All patients completed DHI at a minimum of treatment day and 3 months post SRS. Similar to the study by Stavas et al., no statistical correlation could be found between any of the dose measurements (the mean vestibule dose and maximum to 1 mm^3^ vestibule dose; labyrinth mean dose and maximum to 1 mm^3^ labyrinth dose; and length of nerve receiving treatment dose) and changes in patient-reported dizziness. In contrast, in our study the minimum dose of more than 5 Gy to the vestibule was statistically significantly associated with patient-reported dizziness following SRS. Additionally, mean and maximum doses received by the vestibule were significantly lower in patients who had improved caloric test outcome after SRS. We believe that our study is the first to demonstrate a significant association between vestibular dose and dizziness.

The value of a minimum dose for an organ at risk generally does not have a clinical impact in radiotherapy plan evaluation. However, our findings suggest that limiting the minimum vestibular dose to less than 5 Gy potentially results in fewer patients experiencing dizziness. In our cohort, no dose constraints were given to the vestibule during radiation plan optimization. As a result, the mean and maximum doses to the vestibule are relatively high and similar in all treatment plans. This may help explain why no correlation between dizziness and mean/maximum doses received by the vestibule could be found. Interestingly, patients receiving the lowest mean (1.5 ± 0.7 Gy) and maximum (4 ± 1.5 Gy) doses to the vestibule showed a significant improvement in caloric test in our analysis, although the minimum doses had no effect. Compared to the minimum doses of 5 Gy that had a potential effect on patient-reported dizziness, the mean and maximum doses that positively affected caloric function were much lower.

Risk factors for dizziness other than vestibular dosimetry in our study were consistent with those reported in the literature. Carlson et al. [31] studied patients sporadic VS who had undergone microsurgery, SRS or observation. They reported that female sex, older age, large tumor size, and presence of dizziness or headaches before treatment were associated with long-term dizziness. Our findings suggest that larger tumor volume associated with higher Koos grades and the presence of pre-SRS dizziness were the factors influencing patient-reported dizziness in the univariate analysis.

The study has several limitations due to its retrospective nature. First, one of the most widely used, validated quality of life measurement questionnaires, DHI, was not available for our study. Our patient-reported measurement was coded as a binary outcome, which provides only limited information. Second, a large amount of patient data was missing after treatment, which reduced the sample size and the statistical power. In addition, due to the small number of events (only eight patients experienced dizziness worsening after SRS), the effects of the proportional regression analysis should be interpreted with caution [32]. Third, we reported the early dizziness outcome and factors influencing it and, therefore, our results are limited to short term outcomes and should be extrapolated with care. Forth, we tested only vestibular function at low and middle frequencies, which are not completely reflecting the dynamic range of vestibular function in daily life. Furthermore, we did not fully assess all functions of the vestibule, including ocular VEMPs, which measure utricular function. VEMPs responses can often not be triggered in patients older than 60. Additionally, the measured doses did not include the semicircular canals. Due to the close proximity of the vestibule and the ampulla, a similar dose can be presumed. Finally, there could be inaccuracies in iPlan RT dose for small field dosimetry.

## Conclusions

In this study, we have demonstrated that during a SRS procedure for the treatment of VS, a significant dose of radiation is delivered to the vestibule. We found a significant correlation between the radiation dose received by the vestibule during SRS for VS and the vestibular outcome: 5 Gy and above minimum vestibular doses significantly worsened dizziness following the radiosurgical procedure. Additionally, mean and maximum doses received by the vestibule were significantly lower in patients who had improved caloric function. Further investigations are necessary to validate our findings. We plan to repeat this analysis on a new set of patients using a prospective study design and collecting the dose-volume data of the labyrinth with a precise assessment of the vestibular toxicity.

## Data Availability

The datasets used and/or analysed during the current study are available from the corresponding author on reasonable request.

## Abbreviations

SRS: Stereotactic radiosurgery
VS: Vestibular schwannoma
MR: Magnetic resonance
3D-CISS: Three-dimensional constructive interference in steady-state
cVEMP: Cervical vestibular evoked myogenic potentials
